# What Level Should Preoperative Albumin of Thoracic and Lumbar Tuberculosis Patients Be Reached: A Case-Controlled Study

**DOI:** 10.3389/fnut.2022.740459

**Published:** 2022-04-27

**Authors:** Guanyin Jiang, Yong Zhu, Wei Luo, Wei Zhang, Wanyuan Qin, Yunsheng Ou

**Affiliations:** Department of Orthopedics, The First Affiliated Hospital of Chongqing Medical University, Chongqing, China

**Keywords:** spinal tuberculosis, postoperative hypoalbuminemia, risk factors, scoring scale, recommended value

## Abstract

**Objective:**

To explore the risk factors of hypoalbuminemia in patients with thoracic and lumbar tuberculosis and develop a scoring scale, according to which the patients with thoracic and lumbar tuberculosis were divided into 2 groups to, respectively calculate the perioperative albumin changes and to find out the preoperative albumin recommended value.

**Methods:**

A total of 166 patients with thoracic and lumbar tuberculosis, who underwent spinal focus debridement between January 2012 to May 2020, were identified into 2 groups: with and without postoperative hypoalbuminemia (*n* = 131 and *n* = 35, respectively), recording and analyzing clinical characteristics by multivariate analysis to establish a scoring scale. Using this scale, patients with spinal tuberculosis were divided into a high-risk group and a low-risk group, and then, calculated the average decrease of postoperative albumin in both groups. Combined with the diagnostic threshold of hypoalbuminemia, we proposed the preoperative albumin safe values of the patients with thoracic and lumbar tuberculosis.

**Results:**

A total of 131 of 166 patients experienced postoperative hypoalbuminemia after spinal focus debridement. Multivariate binary logistic regression analysis identified pulmonary tuberculosis (adjusted odds ratio = 0.270, *p* = 0.012), pre-operative serum albumin value (adjusted odds ratio = 0.754, *p* < 0.001), and operation time (adjusted odds ratio = 1.017, *p* = 0.002) as independent risk factors for the occurrence of postoperative hypoalbuminemia in patients with thoracic and lumbar tuberculosis. According to the OR value, the risk factors are assigned to make the scoring scale, the receiver operating characteristic (ROC) curve indicates that postoperative hypoalbuminemia rises when the score is greater than or equal to 4 points. The scoring scale is tested in the derivation set (166 patients) showed: sensitivity-51.9%, specificity-91.4%, and in the validation set (102 patients) showed: sensitivity-63.6% and specificity-86.1%. The perioperative albumin decreased value is 4.71 ± 2.66 g/L in the low-risk group and 8.99 ± 3.37 g/L in the high-risk group (*p* < 0.001).

**Conclusion:**

Complicated with pulmonary tuberculosis, low preoperative albumin value and long operation time can lead to postoperative hypoalbuminemia in patients with thoracic and lumbar tuberculosis. The scoring scale can effectively assist physicians to evaluate whether patients with thoracic and lumbar tuberculosis develop hypoalbuminemia after surgery. The scale is simple and reliable and has clinical guiding significance. For low-risk patients and high-risk patients, preoperative albumin values should reach 40 and 44 g/L, respectively, to effectively avoid postoperative hypoalbuminemia.

## Introduction

Spinal tuberculosis (STB) is common extrapulmonary tuberculosis, accounting for about 50% of osteoarthritis tuberculosis (1). At present, antituberculous drug therapy, combined with surgical treatment, is considered to be the gold standard for the treatment of spinal tuberculosis (2). The debridement of lesions is the key point of spinal tuberculosis therapy, which enhances the control of tuberculosis changes, improves the efficacy of anti-tuberculosis drugs, promotes bone graft fusion, and reduces the risk of recurrence of spinal tuberculosis (3, 4). However, STB debridement has the disadvantages of larger trauma, longer operation time, and more bleeding, considering that 42% of the patients over 60 years old who underwent spinal surgery are malnourished before the operation (5, 6). The possibility of postoperative hypoalbuminemia in patients with STB is significantly higher than in those with a spinal degenerative disease (7–9). Hypoalbuminemia defined as serum albumin less than 35 g/L is commonly considered a representation of malnutrition (10, 11). The ensuing poorer clinical outcomes have been associated with an increased incidence of postoperative complications in a variety of orthopedic surgeries, ranging from spinal fusions to hip fractures, which are closely related to hypoalbuminemia (12–14). In conclusion, patients with spinal tuberculosis are prone to hypoproteinemia after surgery, which can easily lead to various complications and even adverse clinical outcomes. For clinicians, how to avoid postoperative hypoalbuminemia in spinal tuberculosis patients is of great clinical significance. It has been found that preoperative albumin value in patients with total knee arthroplasty is a protective factor for postoperative hypoalbuminemia and the postoperative loss value of serum albumin is closely related to preoperative serum albumin level (15, 16), thus we speculated that the preoperative albumin value of patients with STB may be an important factor in the prevention of postoperative hypoalbuminemia. The objective of our study was to pertinently define the recommended preoperative serum albumin value based on the risk factors of postoperative hypoalbuminemia in patients with STB. In this study, we retrospectively analyzed the clinical characteristics of patients who underwent spinal tuberculosis surgery with or without postoperative hypoalbuminemia and identified three characteristics as independent risk factors of postoperative hypoalbuminemia then established a scoring scale to estimate patients with STB’s incidence of postoperative hypoalbuminemia’s occurrence. Through the ROC curve, we found the diagnostic score of the scoring scale and verified the sensitivity and specificity of the scale in both the derivation group (166 patients) and the validation group (66 patients). Based on the score scale, 166 patients were divided into a low-risk group (≤3 points) and a high-risk group (≥4 points), we calculated the perioperative albumin changing values of the two groups and deduced both groups’ recommended preoperative albumin values by combining the respective average decreased albumin value with the diagnostic threshold of hypoalbuminemia (35 g/L).

## Materials and Methods

All the participants provided their written informed consent to participate in this study before their data were stored in the hospital database and used for research purposes. The work has been reported in line with the STROCSS criteria (17).

### Study Design

This study is a single-center, retrospective, and case-controlled study.

### Study Period

We retrospectively reviewed the records of a total of 166 patients with STB who underwent lesion debridement in our hospital from January 2012 to May 2020 to form the derivation set.

### Study Population

The included patients aged between 14 and 70 had thoracic or lumbar tuberculosis for the first time, and their involved lesions are less than 3 segments. Recorded and analyzed the clinical characteristics including age, gender, height, weight, body mass index (BMI), comorbidities (diabetes, pulmonary tuberculosis), smoking history, drinking history, operation time, operation blood loss, preoperative hemoglobin, preoperative lymphocytes, preoperative albumin, preoperative C-reactive protein (CRP), preoperative erythrocyte sedimentation rate (ESR), and course of the disease.

### Patient Selection

#### Inclusion Criteria

Patients were included when they met the following inclusion criteria: Patients were selected if they met the following inclusion criteria: (i) complete medical records, including general information, preoperative laboratory data, imaging results (MRI and CT), and data on postoperative clinical features, (ii) surgical treatment, (iii) involved lesions less than 3 segments, and (iv) postoperative pathological diagnosis of STB.

#### Exclusion Criteria

Patients were excluded if they met the following exclusion criteria: (i) STB was suspected but not confirmed with pathological examination, (ii) preliminary and pathological diagnosis of a disease like a tumor rather than STB, and (iii) a history of previous STB.

### Validation of the Scoring System

From June 2020 to January 2022, we prospectively included 102 patients with STB who underwent lesion debridement in our hospital to form the validation set. The inclusion criteria and exclusion criteria of the validation set are consistent with the derivation set.

### Statistical Analysis

Measurement data were listed as the mean ± standard deviation (SD) or median (minimum, maximum). Mann-Whitney rank-sum tests or *t*-tests were used to compare the measurement data between the groups. IBM SPSS (version 25.0 for Windows; SPSS, Chicago, IL, United States) was used for logistic regressions to analyze the univariate and multivariate factors for postoperative hypoalbuminemia. Univariate logistic regression analysis was conducted on clinical characteristics to obtain predict factors and then multivariate logistic regression analysis was conducted on these significant factors to confirm the final significant factors. Thresholds for continuous variables (preoperative albumin and operation time) were analyzed using ROC curves. The items of the scoring scale are determined by multivariate logistic regressions, and the weighted score of each item was based on the relative size of the OR value according to the method reported by Kharbanda et al. (18). The ROC curve analysis was used to find the diagnostic threshold of the scale of which the sensitivity and specificity were obtained to access the diagnostic accuracy.

### Ethical Approval

This study was in accordance with the Declaration of Helsinki (as revised in 2013) and was approved by the Institutional Ethics Board of The First Affiliated Hospital of Chongqing Medical University (No. ChiCTR1800019109).

## Results

### Patients Population

Among the total of 166 patients, 131 patients had postoperative hypoalbuminemia and 35 patients did not suffer (Table 1). Seventy-six males and 55 females suffered from postoperative hypoalbuminemia, and 22 male and 13 female did not have postoperative hypoalbuminemia. The mean ages of with postoperative hypoalbuminemia group and without postoperative hypoalbuminemia group were 47.64 ± 1.42 years and 44.43 ± 2.58 years, respectively (Table 1). The clinical characteristics with statistical significance among the with and without postoperative hypoalbuminemia groups are listed as: operation time (*P* < 0.001), operation blood loss (*P* = 0.009), preoperative serum albumin (*P* < 0.001), preoperative CRP (*P* = 0.001), and preoperative ESR (*P* = 0.027) (Table 1).

**TABLE 1:** Perioperative characteristics of 166 patients with thoracic and lumbar tuberculosis.

Characteristics	Postoperative hypoalbuminemia		P
	Yes (*n* = 131)	No (*n* = 35)	
Age (year)	47.64 ± 1.42	44.43 ± 2.58	0.294
BMI (kg/m^2^)	20.96 ± 0.26	22.03 ± 0.67	0.080
Sex (n,%)			0.605
Female	55	13	
Male	76	22	
Diabetes mellitus			0.627
Yes	15	3	
No	116	32	
Pulmonary‘tuberculosis			0.003
Yes	58	6	
No	73	29	
Smoking history (year)	8.50 ± 1.20	6.37 ± 1.79	0.396
Drinking history (year)	5.15 ± 0.99	5.00 ± 1.60	0.945
Operative time (min)	204.17 ± 4.25	169.29 ± 9.44	<0.001
Operative blood loss (ml)	394.89 ± 27.07	246.63 ± 40.18	0.009
Preoperative hemoglobin (g/L)	121.27 ± 1.48	124.89 ± 2.74	0.258
Preoperative lymphocytes (× 10^9^/L)	1.24 ± 0.04	1.41 ± 0.08	0.066
Preoperative serum albumin (g/L)	38.50 ± 0.35	41.37 ± 0.40	<0.001
Preoperative CRP (mg/L)	34.13 ± 3.20	18.16 ± 3.32	0.001
Preoperative ESR (mm/h)	56.24 ± 2.50	44.34 ± 4.41	0.027
Course of disease (month)	12.95 ± 3.05	8.09 ± 2.23	0.421

### Postoperative Serum Albumin Change Trend

**FIGURE 1:**
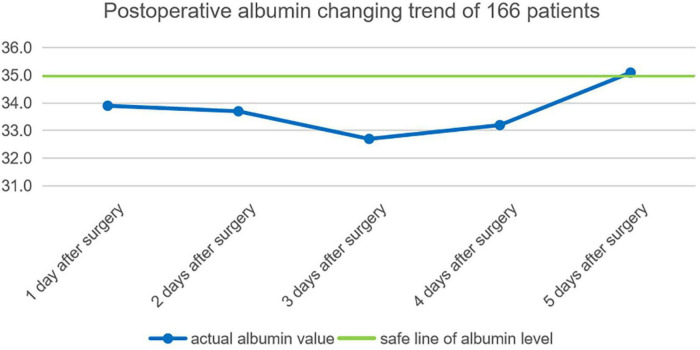
Postoperative albumin changing trend of 166 patients.

By consulting the clinical data, the average albumin of each day among the earliest 5 days after operation of 166 patients are calculated: the first day after the operation is 33.9 ± 3.7 g, second day after the operation is 33.7 ± 3 g, third day after the operation is 32.7 ± 3.9 g, fourth day after the operation is 33.2 ± 3.8 g, fifth day after the operation is 35.1 ± 3.6 g. Postoperative serum albumin change trend 5 days after an operation is shown in Figure 1.

### Results of Univariate and Multivariate Analysis

**FIGURE 2:**
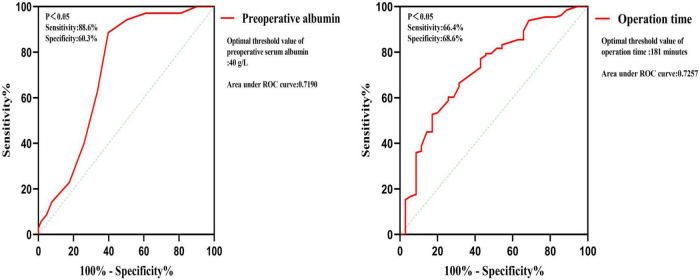
Receiver operating characteristic (ROC) curve of preoperative albumin and operation time.

Univariate logistic regression analysis found that BMI, pulmonary tuberculosis, operation time, operation blood loss, preoperative lymphocytes, preoperative serum albumin, preoperative CRP, and preoperative ESR are risk factors (Table 2). Multivariate logistic regression analysis was used on the above significant risk factors found that pulmonary tuberculosis, preoperative serum albumin, and operation time are independent risk factors of the postoperative hypoalbuminemia’s occurrence (Table 3). ROC curve showed that the cutoff value of preoperative serum albumin was 40 g/L (sensitivity: 88.6%, specificity: 60.3%) and operation time was 181 min (sensitivity: 66.4%, specificity: 68.6%) (Figure 2).

**TABLE 2:** Univariate binary logistic regression analysis of postoperative hypoalbuminemia.

Characteristics	Regression coefficient (β)	Odds ratio (OR)	95% CI	P
Age	0.013	1.013	0.989–1.036	0.293
Sex	–0.203	0.817	0.379–1.761	0.605
BMI	–0.100	0.905	0.807–1.013	0.086*
Diabetes mellitus	–0.322	0.725	0.198–2.660	0.628
Pulmonary tuberculosis	–1.346	0.260	0.101–0.669	0.005*
Smoking history	0.014	1.014	0.983–1.046	0.395
Drinking history	0.001	1.001	0.967–1.036	0.944
Course of disease	0.011	1.011	0.984–1.039	0.436
Operation time	0.017	1.017	1.007–1.026	0.001*
Operation blood loss	0.003	1.003	1.001–1.006	0.009*
Preoperative hemoglobin	–0.013	0.987	0.964–1.010	0.257
Preoperative lymphocytes	–0.692	0.500	0.237–1.057	0.070*
Preoperative serum albumin	–0.247	0.781	0.686–0.889	< 0.001*
Preoperative CRP	0.027	1.028	1.005–1.051	0.016*
Preoperative ESR	0.016	1.016	1.002–1.030	0.030*

* means statistical significance.

**TABLE 3:** Multivariate binary logistic regression analysis of postoperative hypoalbuminemia.

Characteristics	Regression coefficient (β)	Crude odds ratio (OR)	95% CI	P
BMI	–0.059	0.940	0.823–1.080	0.396
Pulmonary tuberculosis	–1.309	0.270	0.097–0.752	0.012*
Operation time	0.017	1.017	1.006–1.028	0.002*
Operation blood loss	0.001	1.001	0.999–1.004	0.295
Preoperative lymphocytes	–0.291	0.748	0.309–1.809	0.519
Preoperative serum albumin	–0.282	0.754	0.648–0.878	< 0.001*
Preoperative CRP	0.003	1.003	0.981–1.027	0.767
Preoperative ESR	<0.001	1.000	0.981–1.019	1.000

* means statistical significance.

### Development of the Scoring Scale

**FIGURE 3:**
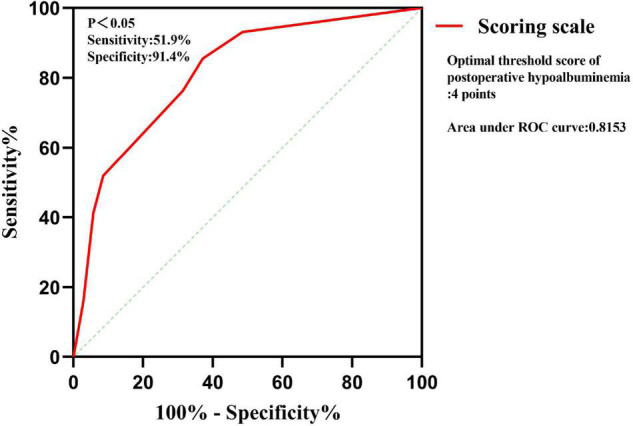
ROC curve of the scoring scale.

Multivariate logistic regression analysis was carried out on the significant findings in univariate analysis and showed 3 clinical characteristics namely pulmonary tuberculosis, preoperative serum albumin, and operation time were significant predictors of STB postoperative hypoalbuminemia’s occurrence (Table 3). According to the OR value, pulmonary tuberculosis (OR = 0.270) was assigned as 1-point, preoperative serum albumin (OR = 0.754) was assigned as 2 points, and operation time (OR = 1.017) was assigned as 3 points (Table 4). ROC curve showed that the diagnostic threshold score of the scoring scale was 4 points (sensitivity: 85.5%, specificity: 62.9%) (Figure 3).

**TABLE 4:** Scoring scale for occurrence of postoperative hypoalbuminemia.

Clinical characteristics	Points
**Pulmonary tuberculosis (in 5 years)**	
Yes	1
No	0
**Preoperative serum albumin (g/L)**	
≤39	2
≥40	0
**Operation time (minute)**	
≥181	3
≤180	0

### Validation of the Scoring Scale

The scoring scale that was made by logistic regression was applied to the 102 cases in the validation set. A comparison of the results of the score scale on the derivation set and validation set was listed in Table 5. Based on the threshold score of 4 points, the sensitivity and specificity of the score for predicting STB postoperative hypoalbuminemia were 51.9 and 91.4% in the derivation set, respectively, 63.6 and 86.1% in the validation set (Table 5).

**TABLE 5:** Comparison of performance of the scoring scale on derivation set and validation set.

		Derivation set			Validation set		
		With POH	Without POH	Total	With POH	Without POH	Total
Clinical diagnosis	With POH	68	63	131	42	24	66
	Without POH	3	32	35	5	31	36
	Total	71	95	166	47	55	102
	Sensitivity (%)	51.9%			63.6%		
	Specificity (%)	91.4%			86.1%		

### Perioperative Serum Albumin Loss Level

**FIGURE 4:**
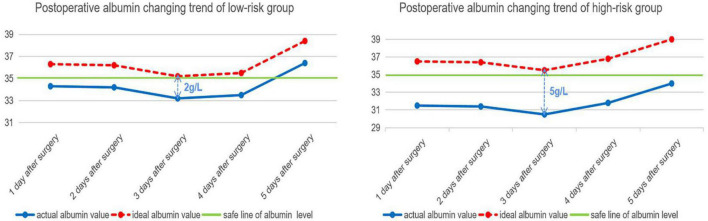
Postoperative albumin changing trend of low-risk group and high-risk group.

Applying the scoring scale on 166 patients to divide into 2 groups, respectively (score ≤3 points group and score ≥4 points group; preoperative albumin value ≤39 g/L groups and ≥40 g/L group) and get the mean value of preoperative serum albumin, postoperative lowest albumin, and postoperative albumin loss, respectively in each group (Table 6). The postoperative albumin changing trend in the earliest 5 days after surgery in both high-risk group and low-risk group has been shown in Figure 4.

**TABLE 6:** Comparison of perioperative albumin changing value divided by scoring scale and preoperative albumin value.

Albumin changing value	Score			Preoperative albumin		
	Score ≤3 points group	Score ≥4 points group	P	≤39 g/L group	≥40 g/L group	P
Preoperative albumin (g/L)	41.37 ± 2.39	38.50 ± 4.00	<0.001	36.06 ± 2.89	42.14 ± 1.85	<0.001
Postoperative lowest albumin (g/L)	36.74 ± 1.85	29.51 ± 3.42	<0.001	29.34 ± 3.88	32.73 ± 4.09	<0.001
Albumin loss (g/L)	4.71 ± 2.66	8.99 ± 3.37	<0.001	6.72 ± 3.90	9.41 ± 4.63	<0.001

### Recommended Preoperative Serum Albumin Value

Combining the diagnostic threshold of hypoalbuminemia and respective average decreased albumin value in the 2 groups, we deduced the recommended preoperative serum albumin value of the patients with STB (Table 7).

**TABLE 7:** Preoperative serum albumin recommended value based on the scoring scale.

Groups	Recommended value
Low risk group (score ≤3 points group)	40 g/L
High risk group (score ≥4 points group)	44 g/L

## Discussion

It is reported that 4.8∼16.8% of patients who underwent spinal surgery are complicated with hypoalbuminemia before operation (19, 20). Hypoalbuminemia, as a common postoperative complication, rises frequently after the spinal operation. Zhang et al. reported that 72.8% of the 602 patients who underwent posterior lumbar fusion, developed hypoalbuminemia after the operation (21). Many studies have shown that hypoalbuminemia is an independent risk factor for wound infection after spinal surgery, which is closely related to the occurrence of perioperative pneumonia, postoperative sepsis, myocardial infarction, and secondary revision operation in both spinal fusion surgery and total hip arthroplasty (22–25). Spinal tuberculosis patients suffer a long course of the disease, are prone to be complicated with malnutrition before operation. The incidence of patients with STB’s postoperative hypoalbuminemia, which is closely related to many severe postoperative complications, is much higher than those with spinal degenerative diseases. It is of great clinical significance to explore the risk factors of hypoproteinemia after operation in patients with spinal tuberculosis. In our study we found that complicated pulmonary tuberculosis, preoperative serum albumin value, and operation time are independent risk factors of postoperative hypoalbuminemia. Based on the independent risk factors, we proposed a simple scoring scale to predict the occurrence of postoperative hypoalbuminemia in patients with thoracic and lumbar tuberculosis. Through the scoring scale we divided 166 patients with thoracic and lumbar tuberculosis into 2 groups, the low-risk group (≤3 points) and the high-risk group (≥4 points), respectively calculated the perioperative albumin changing the value in both groups, then, combined with a diagnostic threshold of hypoalbuminemia. At last, we proposed the recommended values of preoperative serum albumin.

### Independent Risk Factors

#### Pulmonary Tuberculosis

The patients with STB are prone to being complicated with pulmonary tuberculosis. Another epidemiological study of STB based on the same province demographic characteristics reported that 25.7% of 284 patients with STB are complicated with pulmonary tuberculosis (26). Pulmonary tuberculosis is closely linked to malnutrition thus, patients with pulmonary tuberculosis are easier to develop hypoalbuminemia (27). In a study on the nutritional status of patients with tuberculosis, Ddungu et al. reported that 24% of 200 patients with pulmonary tuberculosis had hypoalbuminemia, of which 25% of the patients’ albumin value is less than 25 g/L (28). Not only the albumin, but also these patients’ concentrations of blood hemoglobin, plasma retinol, and plasma zinc are poorer than healthy people (29). The above studies can explain why patients with STB with pulmonary tuberculosis are more likely to occur hypoalbuminemia after surgery.

#### Preoperative Albumin Level

Some studies have shown that postoperative albumin loss is positively correlated with preoperative albumin level, which is considered to be a protective factor for postoperative hypoalbuminemia (15, 16), which is consistent with our research’s results. Our study figured that preoperative albumin was an independent risk factor for postoperative hypoalbuminemia, and the diagnostic threshold value is 40 g/L. Preoperative albumin greater than or equal to 40 g/L is a protective factor for postoperative hypoalbuminemia, for patients with preoperative albumin ≥40 g/L, their postoperative protein loss is more than that of patients with preoperative albumin <40 g/L. Our group holds the view that the albumin loss portion in patients with preoperative albumin greater than or equal to 40 g/L is more than those with preoperative albumin of less than 40 g/L, however, it is not the main cause of postoperative hypoalbuminemia. We proposed a hypothesis and for the convenience to state it, we induced the conception of albumin loss endurance, which means the difference value between the preoperative actual albumin value and the diagnostic value of hypoalbuminemia of 35 g/L. In our hypothesis, patients with preoperative albumin greater than 40 g/L had more albumin loss endurance than patients with preoperative albumin levels lower than 40 g/L, which outnumbers the postoperative excess albumin loss between the two kinds of patients. The advantage of the albumin loss endurance in our hypothesis makes the preoperative albumin value ≥40 g/L a protective factor for postoperative hypoalbuminemia.

The postoperative albumin changing trend in the low-risk group and the high-risk group suggested that there needs to be an improvement of 2 and 5 g/L, respectively, in the low-risk group and high-risk group patients to achieve the safe line of albumin level. The preoperative albumin recommended value proposed by our group is helpful to achieve the ideal postoperative albumin changing trend in the two groups’ patients.

#### Operation Time

In our initial assumption, operative blood loss should be an independent risk factor for postoperative hypoalbuminemia because albumin will be lost from plasma with intraoperative bleeding, albumin was strongly correlated with reliable surrogate parameters of the extent of surgery such as blood loss (5, 30). Unexpectedly, our results suggested that operation time rather than operation blood loss is an independent risk factor for postoperative hypoalbuminemia. According to our analysis, this can be explained by another mechanism of albumin loss in surgical patients, which is called transcapillary escape of albumin. Albumin exudates from the intravascular compartment plasma by dilated capillaries to the tissue, resulting in the decrease of serum albumin after operation, especially under the condition of anesthesia and surgical stress statement (31–33). The operation time of spinal patients, on the one hand, is positively correlated with the operation blood loss, on the other hand, is consistent with patients’ duration of both anesthesia and the surgical stress statement (2, 7–9, 30). Operation time can better synthesize the effects of surgical blood loss and capillaries vasodilation on postoperative albumin loss. The dynamic changes of postoperative albumin suggest that there is a significant increase in postoperative albumin (shown in Figure 1), which may suggest that not all of the reduced albumin is lost with intraoperative bleeding, and albumin lost in the tissue space is re-entered into the blood after the vanishing of anesthesia and the surgical stress statement, thus, resulting in the phenomenon of albumin recovery on the fourth day after operation, which is consistent with our previous explanation. The inclusion of the operation time in the scoring scale brings some difficulties to the evaluation of the patients before the operation. In practice, we should estimate the operation time of preoperative patients according to their specific conditions and combined it with their operation plan of them. This is also a limitation of this study. It also shows that this scale is more suitable for the evaluation of postoperative patients due to the explicit surgical duration.

### Scoring Scale

The proposal of the simple scoring scale is helpful for clinicians to predict whether hypoalbuminemia will occur in patients with thoracic and lumbar tuberculosis after the operation and provide support for whether appropriate treatment measures can be taken to prevent the occurrence of hypoalbuminemia after the operation. Yet, there is no such predictive model of postoperative hypoalbuminemia in patients with STB. Our study is of pioneering significance. The scale is simple and easy to execute and will not add additional workload to clinicians. The specificity of the scoring scale in the derivation set and validation set was, respectively at 91.4% and 86.1%, and both data have confirmed the specificity of the scale is high, which means the patients in the high-risk group authentically have a high incidence to suffer hypoalbuminemia after the operation. The high specificity of the scoring scale brings us new questions like whether patients in the high-risk group can be treated in advance to reduce the occurrence of postoperative hypoalbuminemia, which is the further research direction of our group.

Our score scale also has some unsatisfactory aspects. The sensitivity of the scoring scale in the derivation set and validation set was, respectively 51.9 and 63.6%, which suggests that the missed diagnosis rate of the simple score scale is high. The possibility of hypoalbuminemia after operation in the low-risk group should not be ignored, thus, it is still necessary to pay close attention to the postoperative albumin value of the patients in this group. Due to the limitation of sample size and clinical feature integrity, there is still room for further improvement of our scoring scale, and there may be some independent risk factors for postoperative hypoalbuminemia that have not been revealed.

### Recommended Value of Preoperative Albumin

For more than 50 years, hypoalbuminemia has been associated with increased morbidity and mortality after major abdominal surgeries. Bendersky et al. determined that a threshold preoperative serum albumin of ≥3.9 mg/dL is associated with improved outcomes in elective colorectal surgery patients (34, 35). Similarly, we believed that it is of great clinical significance to explore the appropriate preoperative albumin level in patients with STB, for this consideration, we divided patients with thoracic and lumbar tuberculosis into the low-risk group and high-risk group according to the scoring scale and obtained the postoperative albumin loss values of the two groups. Combined with the diagnostic threshold of hypoalbuminemia: 35 g/L, we proposed the preoperative albumin recommended values for two groups of patients: high-risk group: 44 g/L and low-risk group: 40 g/L. Our study reveals that preoperative albumin level is an independent risk factor for postoperative hypoalbuminemia, which is more likely to occur when the preoperative albumin value is less than 40 g/L. This value coincides with the preoperative albumin recommended value of the low-risk group, which further confirms the rigor of our research results.

For high-risk patients whose preoperative albumin value did not reach the recommended value, the probability of postoperative hypoalbuminemia is very high, so we strongly recommend active treatment. Preoperative nutritional maneuvers play an important role in reducing postoperative impaired healing, morbidities, and mortalities in patients with STB (1, 21). We recommend taking active treatment for this kind of patient, such as supplementing albumin in advance, enhancing the preoperative nutrition, choosing the minimally invasive and short-duration surgical approach. However, for low-risk patients whose preoperative albumin value reached the recommended value, we hold the opinion that it is still necessary to pay close attention to the postoperative albumin change, but there is no need for active preoperative intervention. For some patients whose operation time is hard to predict due to the complex clinical conditions, in consideration of the maximum weight of surgical time on the scoring scale and the safety of postoperative albumin level, we regard the operation time of this kind of patient as greater than 181 min.

## Conclusion

Complicated with pulmonary tuberculosis, low preoperative albumin value and long operation time are three main independent risk factors that can result in postoperative hypoalbuminemia in patients with thoracic and lumbar tuberculosis. The scoring scale proposed by our group can effectively assist the physicians to evaluate whether patients with thoracic and lumbar tuberculosis develop postoperative hypoalbuminemia. The advantage of the scale are only that it is simple and reliable, but also due to its clinical guiding significance. For low-risk patients and high-risk patients, according to our research results, the preoperative albumin value should reach 40 and 44 g/L, respectively, to effectively avoid postoperative hypoalbuminemia.

## Data Availability Statement

The data analyzed in this study is subject to the following licenses/restrictions: The protection of patients’ privacy. Requests to access these datasets should be directed to GJ, 15227175613@163.com.

## Ethics Statement

Written informed consent was obtained from the individual(s), and minor(s)’ legal guardian/next of kin, for the publication of any potentially identifiable images or data included in this article.

## Author Contributions

GJ and YO: conception and design. YZ: provision of study materials of patients. YZ and WL: collection and assembly of data. GJ: data analysis and interpretation. YO: administrative support. All authors contributed to the writing and final approval of the manuscript.

## Conflict of Interest

The authors declare that the research was conducted in the absence of any commercial or financial relationships that could be construed as a potential conflict of interest.

## Publisher’s Note

All claims expressed in this article are solely those of the authors and do not necessarily represent those of their affiliated organizations, or those of the publisher, the editors and the reviewers. Any product that may be evaluated in this article, or claim that may be made by its manufacturer, is not guaranteed or endorsed by the publisher.
